# Alterations of Sublingual Microcirculation in Children With Compensated Type 1 Diabetes Mellitus—An Observational Study

**DOI:** 10.1155/pedi/8811411

**Published:** 2025-09-30

**Authors:** Vlasta Krausova, David Neumann, Lucie Stichova, Jaroslav Skvor, Vlasta Dostalova, Pavel Dostal

**Affiliations:** ^1^Department of Pediatrics, Masaryk Hospital, Krajska Zdravotni, Usti nad Labem, Czech Republic; ^2^Department of Pediatrics, Charles University, Faculty of Medicine in Hradec Kralove, Hradec Kralove, Czech Republic; ^3^Department of Pediatrics, Trutnov Regional Hospital, Trutnov, Czech Republic; ^4^Department of Pediatrics, University Hospital Hradec Kralove, Hradec Kralove, Czech Republic; ^5^Department of Anaesthesiology and Intensive Care Medicine, Charles University, Faculty of Medicine in Hradec Kralove, Hradec Kralove, Czech Republic; ^6^Department of Anaesthesiology and Intensive Care Medicine, University Hospital Hradec Kralove, Hradec Kralove, Czech Republic

**Keywords:** child, sidestream dark-field imaging, sublingual microcirculation, type 1 diabetes

## Abstract

**Introduction:**

Type 1 diabetes is commonly associated with microvascular complications. Sublingual microcirculation examination using sidestream dark-field (SDF) imaging can reflect the situation in visceral microcirculation. The main goals of this observational study were to assess the feasibility of SDF imaging in children with compensated type 1 diabetes, determine selected sublingual microcirculation parameters, and compare them with parameters obtained in healthy children.

**Methods:**

In total 30 children with stable type 1 diabetes without clinical or laboratory signs of microvascular complications were included in the study, 15 males and 15 females in three age categories. Three video clips were recorded using an SDF probe from different parts of the sublingual area and analyzed by software-aided offline analysis.

**Results:**

Videoclips were successfully recorded in all children. Compared with healthy children, the De Backer score (DeBS) in females and total vessel density (TVD), small vessel density (SVD), perfused vessel density (PVD), and perfused SVD (PSVD) in both genders were significantly lower in children with T1D. There were no differences in TVD, SVD, PVD, PSVD, and DeBS between age or gender categories. DeBS correlated with ketonemia; otherwise, no significant relationship was observed between microcirculatory and other recorded parameters.

**Conslusions:**

Sublingual microcirculation examination using SDF imaging is feasible in children with type 1 diabetes. Alteration of sublingual microcirculatory parameters is detectable in children with type 1 diabetes before they show clinical or laboratory signs of any microvascular complication.

**Trial Registration:**

ClinicalTrials.gov identifier: NCT05958264

## 1. Introduction

Type 1 diabetes is commonly associated with microvascular complications [1]. Microcirculation is defined as a network of small vessels (arterioles, capillaries, and venules) with a diameter of less than 100 µm [2]. Microvascular disease occurs predominantly in tissues where glucose uptake is independent of insulin activity [3]. The most common microvascular complications include diabetic kidney disease, eye disease (retinopathy), and nerve disease (neuropathy) [4]. The pathogenesis is not yet fully understood. Many contributive factors, such as low-grade inflammation, epigenetic mechanisms, activation of the renin-angiotensin system, and fluctuations in blood glucose levels, have been described [5, 6]. Altered blood flow and changes in endothelial permeability, extravascular protein deposition, and coagulation result in organ dysfunction [3].

The pathogenesis of microvascular complications starts early in the course of diabetes. It may be present in young people with type 1 diabetes within a few years after diagnosis or at diagnosis in people with youth-onset type 2 diabetes [4]. However, many diabetic patients with early microvascular damage do not have any symptoms for a long time. Thus, annual screening of microvascular complications is recommended by clinical practice guidelines [5, 7].

Sublingual microcirculation was found to reflect the situation of visceral microcirculation [8, 9]. Devices using sidestream dark-field (SDF) imaging emit green light, which is absorbed by hemoglobin of the red blood cells, enabling the visualization of arterioles, capillaries, and venules containing erythrocytes [ 8, 9]. SDF imaging has been used to study changes in microcirculation under various clinical conditions in animal and human studies and is a well-validated modality used to observe microcirculation [8–10]. This method was also recently tested for the early noninvasive detection of diabetic nephropathy [5, 11]. Microvascular alterations, namely loss of the glycocalyx, have been described by several studies recently [12, 13] and can be detected in children with type 1 diabetes mellitus before clinically apparent vascular complications [14].

This study aimed to assess the feasibility of SDF imaging in children with compensated type 1 diabetes, to determine selected parameters of sublingual microcirculation in children with type 1 diabetes with satisfactory treatment of the disease who demonstrate no clinical signs of diabetic microangiopathy, and to compare these parameters with recently published values obtained in healthy children [15].

## 2. Materials and Methods

### 2.1. Study Population

In this prospective, observational study, 30 children with an established diagnosis of type 1 diabetes were included, 15 males and 15 females (Groups M and F) in three different age categories, ten children of younger school age aged 6–10.9 years (Group 1), ten children aged 11–14.9 years (Group 2), and ten adolescents aged 15–18.9 years (Group 3). There was an equal number of boys and girls in all age categories. The study was approved by the Ethics Committee of the Regional Health a.s., Masaryk Hospital in Usti nad Labem, o.z., File Number 310/1.

The study was performed in the Department of Pediatrics, Masaryk Hospital, Krajska Zdravotni, Usti nad Labem, Czech Republic, from 2/2023 to 9/2024. Written informed consent of the child's legal representatives was obtained for all participants. Inclusion criteria included the diagnosis of type 1 diabetes; exclusion criteria included age < 6 and >19 years, acute illness, period of metabolic decompensation and the following 14 days, and known clinical or laboratory signs of microvascular disease based on annual screening as recommended by clinical practice guidelines [7].

### 2.2. Study Protocol

After assessing the patient's medical history and clinical examination to exclude ongoing acute illness, basic anthropometric and selected laboratory parameters and noninvasive blood pressure (BP) and heart rate (HR) were recorded. All measurements were performed in the supine position in a disease-free period, with normal diets, at least 2 h after the last meal in the morning. No premedication or analgesia were used. A total of three video clips was recorded by a single examiner using an SDF probe (MicroScan; MicroVision Medical, Amsterdam, The Netherlands) from different parts of the sublingual area with a minimum length of 20 s.

Recorded parameters included gender, age, height, weight, BP, HR, and blood glucose determined by capillary sampling; ketonemia determined by beta-hydroxybutyric acid concentration from capillary sampling, age of type 1 diabetes diagnosis, duration of type 1 diabetes, last value of glycated hemoglobin, continuous/flash glucose sensor parameters—time in range (TIR), time below range (TBR), and time above range (TAR), glycemic variability in 14 days; and microcirculation parameters: total vascular density (TVD), small vessel density (SVD), perfused vessel density (PVD), perfused SVD (PSVD), DeBacker score (DeBS). A 20 μm cut-off was used to separate small vessels (mostly capillaries) from large vessels (mostly venules).

### 2.3. Data Analysis With Software

Software-aided offline analysis was then used to process the recorded videos. All videos were blindly evaluated by a single researcher trained and experienced in microcirculation evaluation; each video clip's three best and most stable parts were analyzed. Microcirculatory parameters were measured using AVA V3.0 software (AMC, University of Amsterdam, Netherlands).

### 2.4. Power Analysis

A power analysis using an *α* error of 0.05 and *β* error of 0.1 was performed based on previously published normal ranges of microcirculatory parameters in healthy children [15]. In that study, no impact of age on recorded microcirculatory parameters was observed in children aged 3–18 years; however, significantly higher values of several microcirculatory parameters were recorded in females than in males. The sample size was calculated to detect a 20% difference in SVD for each gender separately. This calculation yielded a sample size of 13 females and 15 males. The sample size was increased to 30 subjects to equalize the number of females and males.

### 2.5. Statistical Analysis

Results are expressed as arithmetic mean ± standard deviation according to the normality test results using the D'Agostino–Pearson test. An unpaired t-test was used to assess gender differences in recorded parameters and to compare their values with previously recorded individual values measured in healthy children [15]. An analysis of variance with the Student-Newman-Keuls test was used to compare multiple groups. Correlations between clinical and microcirculatory parameters were expressed using the Kendall rank correlation coefficient. Uncorrected *p*-values are presented; *p*-value < 0.05 was considered significant. MedCalc 7.6.0 (MedCalc Software, Ostend, Belgium) statistical software was used to perform statistical analyses.

## 3. Results

Examination of SDF imaging was attempted in 30 children, and video clips that were useable for offline analysis were successfully recorded in all of them. All children tolerated measurement without premedication or analgesia; no complication occurred during measurement. Anthropometric, laboratory, and macrocirculatory hemodynamic parameters are presented in Table 1.

DeBS in females and TVD, SVD, PVD, and PSVD in both genders were significantly lower in children with type 1 diabetes than in previously measured healthy children [16], Table 2. There were no differences in TVD, SVD, PVD, PSVD, and DeBS between different age or gender categories, Table 3. DeBS value was correlated with ketonemia (*r*_*τ*_ = 0.273, *p*=0.037); otherwise, no significant correlation was observed between microcirculatory and other recorded parameters.

## 4. Discussion

The main finding of this study is the feasibility of SDF imaging in children with type 1 diabetes and the marked difference in sublingual microcirculation parameters between diabetic and healthy children, even before any child showed clinical or laboratory signs of microvascular complications.

These findings are in concordance with other studies that described loss of glycocalyx in patients with type 1 diabetes [13, 14]. The differences in microcirculatory parameters between healthy children and children with type 1 diabetes were more significant in females than males. A higher prevalence of microvascular complications has been reported for adolescent girls compared with boys [16]. In the absence of diabetes, women have a far lower risk of either micro or macrovascular disease compared with men [17]. These differences in vascular biology are probably determined not only by differences in sex steroid levels, mainly of estrogen, but also by gender-specific tissue and sex-specific responses [18]. However, the presence of diabetes confers a greater risk for vascular complications in females compared with males, and potential reasons include both the contribution of sex hormones and sex-specific risk factors [7, 17]. Estrogen exposure, although advantageous with respect to the development of atherosclerotic disease, may, in at risk phenotypes, promote vascular disease [19]. Disordered eating behavior, more common in females in puberty, has been described as a contributing factor leading to the development of microvascular disease [20]. Feminine gender could be also associated with higher emotional stress due to increased resting metabolic amygdala activity associated with increased hemopoietic activity and arterial inflammation [19].

Longer duration of diabetes, older age, and puberty are believed to be risk factors for complications [7]. Our data, however, do not show any difference among different age subgroups.

Apart from a weak correlation between DeBS values and ketonemia, our data showed no relationship between used microcirculatory parameters and recorded anthropometric, laboratory, and macrocirculatory parameters. The children are screened annually for microvascular complications, as clinical practice guidelines recommend [7]. To minimize stress in children, we did not obtain extra venous blood samples for serum lipids and other potential markers [21–23] or urine samples for albuminuria at the time of measurement [24]. Therefore, some possible associations might have been overlooked.

Mechanisms of microcirculatory damage are multiple and to a large extent still unclear. In recent studies, TIR has been associated with diabetes complications [25]. The association was found to be complementary or independent of glycated hemoglobin, further emphasizing the value of TIR [26]. Recently, time in a tight range (TITR) has been proposed for clinical use, using a stricter range of normal glucose levels than TIR [26]. Nonetheless, the relationships between the TIR and the prevalence and risk of diabetic vascular complications and the HbA_1C_ may be negative [27]. We could not identify any association between TIR, TBR, TAR, glycemic variability, or glycated hemoglobin values and used microcirculatory parameters. This finding suggests that the glycemic control metrics currently in use might not be sensitive enough for the early detection of microcirculatory damage. Alternatively, it is possible that the duration of glycemic exposure or unmeasured fluctuations in blood sugar levels contribute more significantly to the development of vascular complications than is currently acknowledged. Other pathways, besides hyperosmolarity and hyperglycemia, that impair the microcirculation in diabetes, all involved in the maintenance of vascular homeostasis and permeability, are also studied [28, 29]. While glycemic control can have uncertain results in controlling macrovascular complications, it still remains the most effective strategy to prevent them [28].

Our study has several limitations. All children were Caucasians. For logistical reasons, historical controls, rather than a concurrently enrolled control group, were used in this study. Although it represents a potential limitation of this study, the methodology used was identical for both historical controls and the studied children with type 1 diabetes. More subtle details might have been noticed with more enrolled participants, even though the group size was chosen according to a power analysis. Software-aided offline analysis using AVA 3.0 software was used to determine microcirculatory parameters. Therefore, results could differ compared to the results obtained using other software analysis tools. Our data do not allow any conclusion regarding the possible relationship between the values of microcirculatory parameters and individual risk of clinical manifestation of microvascular complications.

## 5. Conclusions

Sublingual microcirculation examination using SDF imaging is feasible in children with type 1 diabetes. Alteration of sublingual microcirculatory parameters is detectable in children with type 1 diabetes before they show clinical or laboratory signs of any microvascular complication. Following this cohort of patients over time will be crucial to determine the association between these early sublingual microcirculatory changes and the development of clinical nephropathy, retinopathy, or neuropathy.

## Figures and Tables

**Table 1:** Anthropometric, baseline macrocirculatory, and selected laboratory parameters.

Variable	Group 1 6–10.9 years n = 10	Group 2 11–14.9 years n = 10	Group 3 15–18.9 years n = 10
Age (years)	8.0 ± 1.55	12.9 ± 1.14	16.8 ± 0.98
Male gender, *n* (%)	5 (50%)	5 (50%)	5 (50%)
Height (cm)	136.0 ± 9.86	166.5 ± 8.04	170.9 ± 5.69
Weight (kg)	35.4 ± 10.17	59.7 ± 11.07	65.2 ± 11.26
Sensor (%)	70%	100%	100%
Onset of T1D (years)	5.2 ± 2.19	8.0 ± 3.81	8.4 ± 5.33
Duration of T1D (years)	2.8 ± 2.02	5.0 ± 3.58	8.3 ± 5.89
Hb1Ac (mmol/mol)	53.6 ± 9.06	50.9 ± 8.02	52.1 ± 9.69
TIR (%)	68.1 ± 18.62	78.0 ± 8.96	73.8 ± 21.44
TBR (%)	2.7 ± 1.03	1.7 ± 1.00	2.6 ± 3.13
TAR (%)	29.3 ± 18.58	20.3 ± 8.94	24.0 ± 21.12
GV (%)	35.2 ± 3.35	31.8 ± 4.46	33.7 ± 7.68
Glycemia (mmol/L)	6.7 ± 1.01	8.1 ± 2.02	7.5 ± 2.10
Ketonemia (mmol/L)	0.10 ± 0.08	0.13 ± 0.05	0.12 ± 0.04
Heart rate (/min)	86.9 ± 13.84	82.4 ± 10.41	85.2 ± 11.82
SAP (mmHg)	101.1 ± 13.14	125.7 ± 16.55	125.3 ± 9.81
DAP (mmHg)	74.9 ± 15.01	77.7 ± 7.91	81.0 ± 7.08
MAP (mmHg)	83.6 ± 14.22	93.7 ± 7.72	95.8 ± 6.85

Abbreviations: DAP, diastolic arterial pressure; GV, glycemic variability; MAP, mean arterial pressure; SAP, systolic arterial pressure; T1D, type 1 diabetes; TIR, time in range; TBR, time below range; TAR, time above range.

**Table 2:** Microcirculatory parameters in healthy children and children with type 1 diabetes mellitus.

Variable	Group M	Controls M	*p*-Value	Group F	Controls F	*p*-Value
TVD (mm/mm^2^)	10.16 ± 1.46	11.93 ± 2.27	0.013	10.76 ± 1.00	13.68 ± 1.89	<0.0001
SVD (mm/mm^2^)	7.37 ± 2.26	9.11 ± 2.43	0.039	7.92 ± 1.52	11.05 ± 2.25	0.0001
PVD (mm/mm^2^)	9.93 ± 1.35	11.37 ± 2.27	0.037	10.64 ± 0.95	13.38 ± 1.88	<0.0001
PSVD (mm/mm^2^)	7.17 ± 2.12	8.85 ± 2.39	0.038	7.78 ± 1.60	10.94 ± 2.24	0.0001
DeBS (1/mm)	6.84 ± 1.01	7.61 ± 1.35	0.072	7.04 ± 0.85	8.76 ± 1.38	<0.0001

Abbreviations: DeBS, DeBacker's score; PSVD, perfused small vessel density; PVD, perfused vessel density; SVD, small vessel density; TVD, total vessel density.

**Table 3:** Microcirculatory parameters in different age groups.

Variable	Group 1 6–10.9 years *N* = 10	Group 2 11–14.9 years *N* = 10	Group 3 15–18.9 years *N* = 10	*p*-Value
TVD (mm/mm^2^)	10.37 ± 0.76	10.41 ± 1.06	10.60 ± 1.86	0.921
SVD (mm/mm^2^)	7.52 ± 1.45	7.37 ± 1.74	8.05 ± 2.35	0.725
PVD (mm/mm^2^)	10.23 ± 0.70	10.22 ± 0.96	10.40 ± 1.66	0.936
PSVD (mm/mm^2^)	7.39 ± 1.48	7.19 ± 1.70	7.85 ± 2.20	0.737
DeBS (1/mm)	6.92 ± 0.50	7.07 ± 0.77	7.13 ± 1.12	0.869

Abbreviations: DeBS, DeBacker's score; PSVD, perfused small vessel density; PVD, perfused vessel density; SVD, small vessel density; TVD, total vessel density.

## Data Availability

The full data are available from the corresponding author upon request.

## Nomenclature

DeBS: DeBacker score
HR: Heart rate
SDF: Sidestream dark-field
PSVD: Perfused small vessel density
PVD: Perfused vessel density
SVD: Small vessel density
TAR: Time above range
TBR: Time below range
TIR: Time in range
TITR: Time in the tight range
TVD: Total vessel density.
